# A Novel Binary Slime Mould Algorithm with AU Strategy for Cognitive Radio Spectrum Allocation

**DOI:** 10.1007/s44196-021-00005-0

**Published:** 2021-09-20

**Authors:** Ling Li, Tien-Szu Pan, Xiao-Xue Sun, Shu-Chuan Chu, Jeng-Shyang Pan

**Affiliations:** 1 College of Computer Science and Engineering, Shandong University of Science and Technology, Qingdao, 266590 China; 2 Department of Electronic Engineering, National Kaohsiung University of Science and Technology, Kaohsiung, Taiwan; 3 College of Science and Engineering, Flinders University, 1284 South Road, Clovelly Park, 5042 SA Australia; 4 Department of Information Management, Chaoyang University of Technology, Taichung, Taiwan

**Keywords:** Slime mould algorithm, Binary optimization, Spectrum allocation, Cognitive radio

## Abstract

Spectrum has now become a scarce resource due to the continuous development of wireless communication technology. Cognitive radio technology is considered to be a new method to solve the shortage of spectrum resources. The spectrum allocation model of cognitive radio can effectively avoid the waste of spectrum resources. A novel binary version of slime mould algorithm is proposed for the spectrum allocation model to solve the spectrum allocation scheme. In addition, adding unselected factors strategy can make the approach find a better solution. Compared with other algorithms, the novel binary slime mould algorithm and the strategy of adding unselected factors proposed in this paper have a good performance in spectrum allocation. The resulting spectrum allocation scheme can achieve efficient use of network resources.

## Introduction

Wireless communication technology has developed rapidly in recent years. The wireless network has become an essential part of daily life. Growth in demand for wireless communication services makes the spectrum become a scarce resource. The allocation of spectrum resources is related to the development of radio technology. At the current stage, the spectrum resources implement a static allocation strategy. This static strategy avoids interference between communication systems to a certain extent. But when authorized users do not use the spectrum, the corresponding spectrum resources will be wasted. To solve the problem of low spectrum utilization, the concept of cognitive radio technology [1, 2] is proposed. Its idea is to enable unauthorized wireless communication devices to actively discover and make use of the vacancy of dedicated licensed spectrum bands.

Cognitive radio can perceive the external radio environment, capture free spectrum resources, count and analyze external environmental changes. It can modify the wireless transmission parameters according to the dynamic setting of the environment so that cognitive radio equipment adopts different transmission technologies for data send. There are mainly three essential cognitive radio technologies: spectrum sensing technology, dynamic spectrum allocation technology, and power control technology. This paper primarily studies the spectrum allocation technology and achieves efficient utilization of spectrum resources through a reasonable spectrum allocation.

The spectrum allocation mainly studies how to share the spectrum band among unlicensed cognitive users after a free spectrum band is discovered. Many studies have proposed relevant spectrum allocation models, including game theory [3], pricing and auction mechanisms [4], and graph coloring [5]. The spectrum allocation problem is actually an NP-hard problem [6]. It is impractical to get its optimal solution through exhaustive search. Because the metaheuristic algorithm performs better on many problems, many scholars use it to solve the problem of spectrum allocation. Zhijin Zhao et al. [7] introduced genetic algorithm and particle swarm algorithm (PSO) to the spectrum allocation problem and achieved good results. Abdelsalam HM et al. [8] proposed an enhanced particle swarm optimization for spectrum assignment in cognitive radio networks. However, these methods have problems such as high computational complexity or single search direction in the later stage of convergence. More effective optimization approaches need to be explored.

The metaheuristic algorithm has two main stages, exploration and exploitation. The exploration stage can search in the entire solution space to find potential solutions to avoid local optima. In the exploitation stage, a better solution is found near the obtained solution. A good metaheuristic algorithm should maintain the balance between two stages. The swarm intelligence algorithm is part of the metaheuristic algorithm [9, 10]. It is a population-based intelligent algorithm that simulates the population behavior in the natural environment. Genetic algorithm (GA) [11, 12] is the earliest swarm intelligence algorithm. GA is a kind of evolutionary algorithms [13–15]. It searches for the optimal solution by simulating the natural evolution process of organisms. PSO [16–23] simulates the behavior of birds looking for a destination during the migration process and changes the position through the velocity vector. Grey wolf optimization (GWO) [24, 25] imitates the hierarchical structure and hunting behavior of wolves. Quasi-affine transformation evolution (QUATRE) algorithm [26–28] is a co-evolution framework for quasi-affine transformation, which can perform a statistical and probabilistic search. Due to the complexity of the actual problem, multi-objective optimization has gradually developed [29–32]. Multiple objective functions need to be optimized at the same time. Non-dominated Sorting Genetic Algorithm II (NSGA II) is a dynamic multi-objective algorithm that effectively reduces the computational complexity and can maintain a better spread of solutions [33–36].

Many standard optimization algorithms cannot be directly used for discrete problems. In the optimization of 0-1 knapsack, spectrum allocation, and feature selection, it is necessary to search in the binary space. Therefore, the binary versions are required for solving these discrete problems. The binary particle swarm optimization (BPSO) [37, 38] uses the SF (sigmoid function) method to discretize the continuous value of the speed so as to complete the judgment of the new position as 0 or 1. The binary grey wolf algorithm(BGWO) [39] was first proposed by Zawbaa et al. to solve the feature selection problem. Because GA can directly use binary coding methods for genes, they can be used to optimize discrete problems [40]. Quantum genetic algorithm (QGA) [7, 41] includes quantum computing and genetic evolution. Like GA, QGA can also be used in binary optimization problems.

Although the metaheuristic algorithm has achieved great success in many aspects, it cannot guarantee that good results can be achieved on all optimization problems. Therefore, many researchers are committed to proposing new optimization algorithms [42–45]. Slime mould algorithm (SMA) is a new swarm intelligence algorithm proposed by Shimin Li et al [46]. SMA [47–49] simulates the behavior of Physarum polycephalum using the biological shock mode to search food in nature. And the weight is used to simulate the positive and negative feedback during the foraging process. Shimin Li et al. confirmed that compared with other optimization algorithms, SMA has good performance in exploration and exploitation on unimodal and multimodal functions. According to the advantages and limitations of each meta-heuristic algorithm, some scholars propose to combine multiple optimization methods to achieve better solutions [50–53]. The combination of SMA and adaptive guided differential evolution (AGDE) effectively enhances the local search capabilities of agents and helps avoid premature convergence [54]. In Ref. [55], SMA is combined with whale optimization algorithm (WOA) to extract the region of interest containing COVID-19 features in the X-ray images, to achieve the goal of improving the accuracy of image classification. Binary slime mould algorithm (BSMA) was first proposed by Abdel-Basset et al. to solve the feature selection problem [56]. They also proposed three improved algorithms for BSMA based on the attacking-feeding strategy and the two-phase mutation [57]. However, when the original position update rule is transferred to the binary space, it is easy to fall into the local optimum to a certain extent. Therefore, we propose a novel binary version of slime mould algorithm to solve the spectrum allocation in cognitive networks. The main work of this paper is listed as follows;

1. A novel binary slime mould algorithm is proposed to solve the spectrum allocation problem.
2. Introduce a new transfer function and add it to the performance comparison of S-shaped and V-shaped transfer functions. The transfer function with the best performance is selected.
3. A new adding unselected factors strategy (AU strategy) mutates the poorer solutions in the population.
4. Compare the two proposed approaches with the existing binary versions of the SMA.
5. Compare with the optimization algorithms used to solve the spectrum allocation to verify the performance of the two proposed approaches.

The rest of this paper is organized as follows: Section 2 describes the SMA, BSMA, and spectrum allocation models. Section 3 proposes a novel binary SMA and a mutation strategy for solutions with poor performance. Section 4 verifies the proposed novel binary SMA and mutation strategy. Section 5 draws conclusions and puts forward some inspirations for future work.

## Preliminaries

This section briefly describes the standard SMA and BSMA. The spectrum allocation model and the fitness functions are also introduced.

### Slime Mould Algorithm

The working process of SMA includes three stages: approaching food, wrapping food, and grabbing food.

(1) Approaching food

The slime mould approaches food according to the smell in the air. The following formula is proposed to express approach behavior.

1 $$\begin{aligned} \varvec{\overrightarrow{X(t+1)}}=\left\{ \begin{array}{lr} \varvec{\overrightarrow{X_{b}(t)}}+\varvec{\overrightarrow{vb}}\cdot (\varvec{\overrightarrow{W}} \cdot \varvec{\overrightarrow{X_{A}(t)}}-\varvec{\overrightarrow{X_{B}(t)}}),r<p \\ \varvec{\overrightarrow{vc}} \cdot \varvec{\overrightarrow{X(t)}},r \ge p \end{array} \right. \end{aligned}$$

where $\varvec{\overrightarrow{vb}}$ is a parameter in the range of $[-a, a]$. $\varvec{\overrightarrow{vc}}$ is a parameter that linearly decreases from 1 to 0, *t* represents the current iterative process. $\varvec{\overrightarrow{X_{b}} }$ represents the closest individual position of the current process to the target. Equation (1) indicates that the slime mould updates the position of the search individual according to the currently obtained optimal position $\varvec{\overrightarrow{X_{b}} }$, and the weight vector $\varvec{\overrightarrow{W}}$. $\varvec{\overrightarrow{vc}}$ and $\varvec{\overrightarrow{vb}}$ can change the position of the individual. $\varvec{\overrightarrow{X_{A}} }$ and $\varvec{\overrightarrow{X_{B}} }$ represent two individuals randomly selected from the population. The definition of *p* is given in Eq. (3). When $r<p$, the mould is in the global search and gradually moves closer to the best position found. When $r\ge p$, the search range of slime mould is reduced, and it is in the state of local search.

$\varvec{\overrightarrow{W}}$ represents the weight of slime mould, defined by Eq. (2), which represents the influence of the food concentration of slime mould during its movement. The slime mould mainly relies on the propagation wave generated by the biological oscillator to change the cytoplasmic flow. The higher the concentration of nearby food, the stronger the propagation wave generated by the oscillator, and the larger the width of the vein structure. This feature ensures that the slime mould gets enough nutrition in the area. To a certain extent, it can be said that $\varvec{\overrightarrow{W}}$ simulates the biological oscillator of slime mould.

2 $$\begin{aligned} \overrightarrow{W(SmellIndex(l))}=\left\{ \begin{array}{lr} 1+r\cdot log\left( \frac{bF-S(i)}{bF-wF} +1 \right) , condition \\ 1-r\cdot log\left( \frac{bF-S(i)}{bF-wF} +1 \right) , others \end{array} \right. \end{aligned}$$

where *bF* represents the best fitness value in the current iterative process. *wF* represents the worst fitness value in the current iterative process. *r* is a random number uniformly distributed in the range of [0, 1], which simulates the uncertainty of the slime mould contraction pattern in the natural environment. *SmellIndex* represents the sorted result of the fitness value of slime mould. *l* represents the position of the individual number *i* in *SmellIndex*. *condition* represents the individuals ranked in the top half of the *SmellIndex*.

*p* is defined as follows:

3 $$\begin{aligned} p=tanh( \mid S(i)-DF \mid ), \end{aligned}$$

where *p* represents the gap between an individual and the current best fitness value, used to determine the position update strategy, *DF* represents the best fitness obtained in the iterations so far.

*a* is defined as follows:

4 $$\begin{aligned} a=arctanh\left( 1-\frac{t}{maxiter}\right) , \end{aligned}$$

where *t* represents the current iteration number, *a* gradually decreases as the number of iterations increases. *maxiter* represents the maximum number of iterations in the search process.

(2) Wrapping food

The rule for updating the position of slime mould is as follows:

5 $$\begin{aligned} \varvec{\overrightarrow{X(t+1)}} =\left\{ \begin{array}{lr} rand \cdot (\varvec{\overrightarrow{UB}}-\varvec{\overrightarrow{LB}})+\varvec{\overrightarrow{LB}}, rand<z \\ \varvec{\overrightarrow{X_{b}(t)}}+\varvec{\overrightarrow{vb}}\cdot (\varvec{\overrightarrow{W}} \cdot \varvec{\overrightarrow{X_{A}(t)}}-\varvec{\overrightarrow{X_{B}(t)}}),r<p \\ \varvec{\overrightarrow{vc}} \cdot \varvec{\overrightarrow{X(t)}},r \ge p\\ \end{array} \right. \end{aligned}$$

where *rand* and *r* are two different random values in the range of [0, 1]. The value of *z* is related to maintaining the balance of exploitation and exploration. After experimental analysis, it is found that the optimization performance of the algorithm is better when $z=0.03$ [46]. $\varvec{\overrightarrow{LB}}$ and $\varvec{\overrightarrow{UB}}$ represent the lower and upper bounds of the search space.

(3) Grabbing food

$\varvec{\overrightarrow{vb}}$ and $\varvec{\overrightarrow{vc}}$ simulate the selection behavior of slime mould. In order to find a better food source, even if the slime mould finds a target with a higher food concentration, it will disperse parts of the organism to search for other areas instead of focusing on one food source. The value of $\varvec{\overrightarrow{vb}}$ is within $[-a, a]$, which helps to avoid local optima.

The pseudo-code of the continuous SMA is shown in Algorithm 1. 
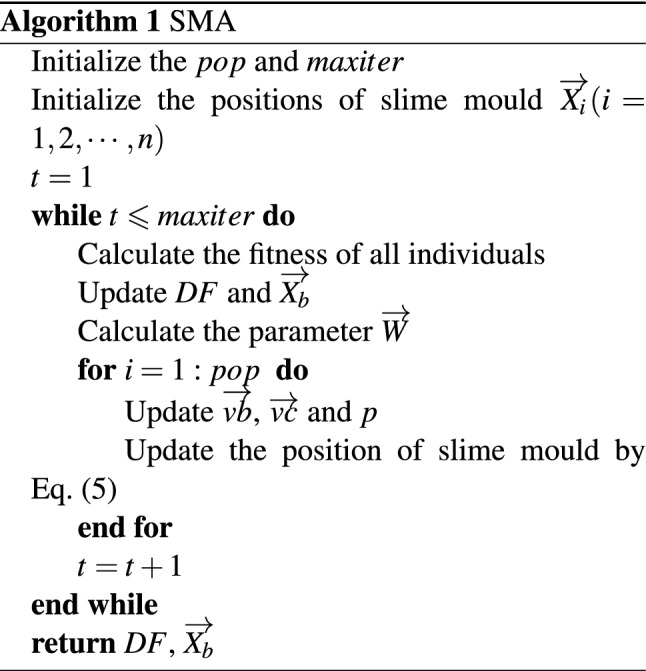


### Binary Slime Mould Algorithm

In SMA, the value of each dimension of the solution can only be continuous. Due to the need to solve the optimization problem of the binary space, BSMA is proposed. The pseudo-code of the BSMA is shown in Algorithm 2. Besides, three binary versions of SMA have also been proposed, namely BSMA with two-phase mutation (TMBSMA), BSMA with attacking-feeding strategy (AFBSMA), and BSMA with a combination of two-phase mutation and attacking-feeding strategy (FMBSMA).

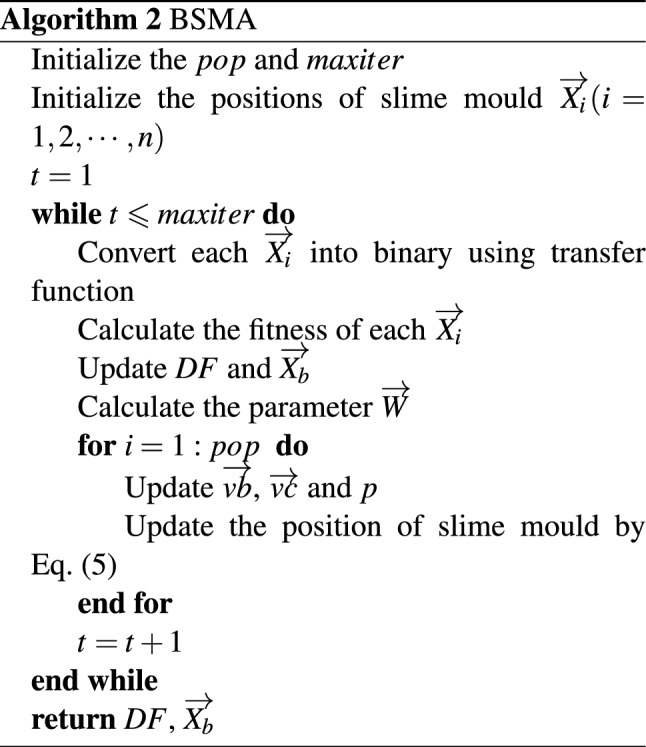



When using Eq. (5) to update the position, there are only four choices for $X_{A}^{d}$ and $X_{B}^{d}$ in the binary search space, which are (0,0), (0,1), (1,0) and (1,1). Without the transfer function conversion, the value or range of $X_{i}^{d}$ has four cases in Table 1.

**Table 1:** Four cases of $X_{i}^{d}$ value when $r<p$ in Eq. (5)

$(X_{A}^{d},X_{B}^d)$	$X_{i}^{d}$	The value of $X_{i}^{d}$	
		when $X_{b}^{d}=1$	when $X_{b}^{d}=0$
(0,0)	$X_{b}^{d}$	1	0
(0,1)	$X_{b}^{d}-vb^{d}$	[− 2.45,4.45]	[− 3.45,3.45]
(1,0)	$X_{b}^{d}+vb^{d} \cdot W(i)$	[− 3.485,5.485]	[− 4.485,4.485]
(1,1)	$X_{b}^{d}+vb^{d} \cdot (W(i)-1)$	[− 0.035,2.035]	[− 1.035,1.035]

^1^ When maxiter is 500, the range of vb is [− 3.45,3,45]. Taking the fitness function represented by Eq. (7) as an example, the value range of *W*(*i*) is roughly [0.7,1.3]

When using the transfer function for conversion, whether it is S-shaped or V-shaped, if $X_{b}^{d}=1$, then $X_{i}^{d}$ will have a high probability of taking 1. This makes the algorithm easy to fall into the local optimum to a certain extent.

### The Spectrum Allocation Model

Spectrum allocation is a crucial technology of cognitive networks. The main goal of spectrum allocation is to allocate spectrum to cognitive users to reduce the waste of spectrum resources while avoiding interference to authorized users.

Through the four matrices of channel availability matrix, channel reward matrix, interference constraint matrix, and conflict-free channel assignment matrix in Refs. [7], reasonable allocation of spectrum resources can be realized. Assuming that there are *N* cognitive users in the wireless network and *M* available spectrum bands are perceived. The four spectrum allocation matrices are defined as follows:

(i) The channel availability matrix $\varvec{L}=\{ l_{n,m}\mid l_{n,m}\in \{0,1\} \}_{N \times M}, 1\le n\le N, 1\le m \le M.\quad l_{n,m}$ represents the ownership of available channels for cognitive users. $l_{n,m}=1$ indicates that cognitive user *n* can use the channel numbered *m*. $l_{n,m}=0$ indicates that it cannot be used.
(ii) The channel reward matrix $\varvec{B}=\{b_{n,m} \}_{N \times M},b_{n,m}$ represents the benefits that cognitive user *n* obtains on channel *m*. Network benefits can be expressed by many factors such as the maximum network traffic, maximum throughput, and spectrum utilization. In this paper, network bandwidth is used to quantify the benefits achieved.
(iii) The interference constraint matrix $\varvec{C}=\{ c_{n,k,m} \mid c_{n,k,m} \in \{ 0,1 \}\}_{N \times N \times M}$, where n and k both indicate cognitive users. $c_{n,k,m}$ indicates whether the simultaneous use of the m-th channel by the n-th and k-th cognitive users will cause interference. If the value is 1, it means that two users will interfere with each other.
(iv) The conflict-free channel assignment matrix $\varvec{A}=\{ a_{n,m}\mid a_{n,m} \in \{0,1\} \}_{N \times M}$, $a_{n,m}$ indicates whether channel *m* can be allocated to cognitive user *n* under the interference constraint matrix $\varvec{C}$. If the value is 1, it means channel *m* can be assigned. $\varvec{A}$ must satisfy the following constraints: $c_{n,k,m}=1 \cap (a_{n,m}+a_{k,m} \le 1)$.

**Fig. 1:**
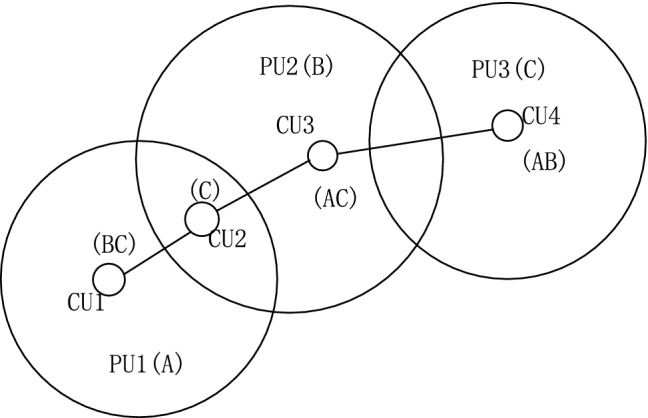
The topology of the cognitive network at a certain moment

Figure 1 shows the topology of the cognitive wireless network at a certain moment. The channel is represented by {A, B, C}, and the cognitive user is marked by {CU1, CU2, CU3, CU4}. Three authorized users are represented by {PU1, PU2, PU3}. The arc represents the communication coverage of authorized users. Suppose that at this moment, the authorized user PU1 owns channel A, PU2 owns B, and PU3 owns C. Since CU1 is located in the communication coverage area of PU1, if the cognitive user uses channel A, it will interfere with PU1. The available channel for CU1 is {B, C}. Similarly, the available channel of CU2 is {C}, the available channel of CU3 is {A, C}, and the available channel of CU4 is {A, B}. Therefore, the specific information of the channel available matrix $\varvec{L}$ can be obtained.

6 $$\begin{aligned} \varvec{L}= \begin{pmatrix} 0 &{} 1 &{}1 \\ 0 &{} 0 &{}1\\ 1&{}0&{}1\\ 1&{}1&{}0\\ \end{pmatrix} \end{aligned}$$

### Fitness Function

According to the final conflict-free channel assignment matrix $\varvec{A}$ and reward matrix $\varvec{B}$, the benefits obtained by each user can be obtained. Then, the network benefits that the user with number *n* can obtain are $\sum _{m=1}^{M}a_{n,m}\cdot b_{n,m}$.

By accumulating the reward of all users, the Max-Sum-Reward (MSR) of the current network after the completion of the spectrum allocation can be obtained.

7 $$\begin{aligned} MSR=max\sum _{n=1}^{N}\sum _{m=1}^{M}a_{n,m}\cdot b_{n,m}. \end{aligned}$$

In order to measure the fairness of the benefits obtained by each user, it can be expressed by the Max-Proportional-Fair (MPF).

8 $$\begin{aligned} MPF=max\left[ \prod _{n=1}^{N}\left( \sum _{m=1}^{M}a_{n,m}\cdot b_{n,m}+10^{-4}\right) \right] ^{\frac{1}{N}}. \end{aligned}$$

In this paper, Eqs. (7) and  (8) are used as fitness functions.

## The Proposed Novel Binary Slime Mould Algorithm

In SMA, the algorithm only searches in a continuous space. But there are some special problems whose search space is binary, such as feature selection, 0-1 knapsack, and spectrum allocation problem. In this section, a novel binary version of the SMA (NBSMA) is proposed to solve the spectrum allocation problem. NBSMA can improve the disadvantage of falling into the local optimum in the BSMA update rules.

### Transfer Function

The transfer function maps continuous values to [0,1] and then converts them to 0 or 1 according to the probability. In the optimization of binary problems, the transfer function is crucial. Even if the solution is binary in the initial state, after a series of processing and conversion, non-binary situations will inevitably occur. Therefore, using the transfer function is a very effective method.

S-shaped and V-shaped functions are currently the most common transfer functions. Mirjalili et al. [58] compared the performance of S-shaped function and V-shaped function on binary particle swarm optimization. Abdel-Basset et al. [56] compared eight transfer functions on the proposed BSMA algorithm and proved that the S4 and V1 transfer functions in Table 2 are the two most effective functions for feature selection. In this section, S5 is introduced as a new transfer function. The S5 transfer function has been used in the binary grey wolf optimization algorithm to solve feature selection [39]. This paper compares the performance of the three transfer functions of S4, S5, and V1. The transfer function with the best performance is selected and used in subsequent comparisons. The details and results of the experiment are shown in Sect. 4.

**Table 2:** Details of the transfer function

Abbreviation	Transfer function
S1	$F(x)=\frac{1}{1+e^{(-2x)}}$
S2	$F(x)=\frac{1}{1+e^{(-x)}}$
S3	$F(x)=\frac{1}{1+e^{(-\frac{x}{2})}}$
S4	$F(x)=\frac{1}{1+e^{(-\frac{x}{3})}}$
S5	$F(x)=\frac{1}{1+e^{-10(x-0.5)}}$
V1	$F(x)=\vert erf \left( \frac{\sqrt{2}}{\pi }x\right) \vert$
V2	$F(x)=\vert tanh(x) \vert$
V3	$F(x)=\frac{x}{\sqrt{1+x^{2}}}$
V4	$F(x)=\vert \frac{2}{\pi }arctan( \frac{\pi }{2} x) \vert$

### A Novel Binary Slime Mould Algorithm (NBSMA)

In the NBSMA, the position update rule is shown in Eq. (9):

9 $$\begin{aligned} X_{i}^{d}(t+1)=\left\{ \begin{array}{lr} x_1, rand<z \\ x_2, r<p\\ x_3, r\ge p\\ \end{array} \right. \end{aligned}$$

where $x_{1}$, $x_{2}$ and $x_{3}$ can be given by Eqs. (10),  (11) and  (12), respectively.

10 $$\begin{aligned} x_{1}=\left\{ \begin{array}{lr} 1, rand>0.5 \\ 0, rand \le 0.5 \end{array} \right. \end{aligned}$$

where *rand* represents a random number in the range [0, 1].

11 $$\begin{aligned} x_{2}=\left\{ \begin{array}{lr} 1-X_{b}^{d}, F(vb^{d} \cdot (W(i) \cdot X_{A}^{d}-X_{B}^{d})) \ge rand\\ X_{b}^{d}, others \end{array} \right. \end{aligned}$$

where $X_{b}^{d}$ represents the value of the $\varvec{\overrightarrow{X_{b}}}$ in the d-th dimension. *rand* represents a random number in the range [0, 1]. $vb^{d}$ is a random number in the range of $[-a,a]$. *W*(*i*) is given by Eq. (2). $\varvec{\overrightarrow{X_{A}}}$ and $\varvec{\overrightarrow{X_{B}}}$ represent two individuals randomly selected from the population. *F*(*x*) represents a transfer function, which is used to convert continuous values into values in the range [0, 1]. $x_{3}$ is defined as follows:

12 $$\begin{aligned} x_{3}= \left\{ \begin{array}{lr} 1-X_{i}^{d}, F(vc^{d} \cdot X_{i}^{d})>rand\\ X_{i}^{d}, others \end{array} \right. \end{aligned}$$

where *rand* is a random number in the range [0,1], and *i* represents the i-th individual in the population. The pseudo-code of the NBSMA is shown in Algorithm 3.

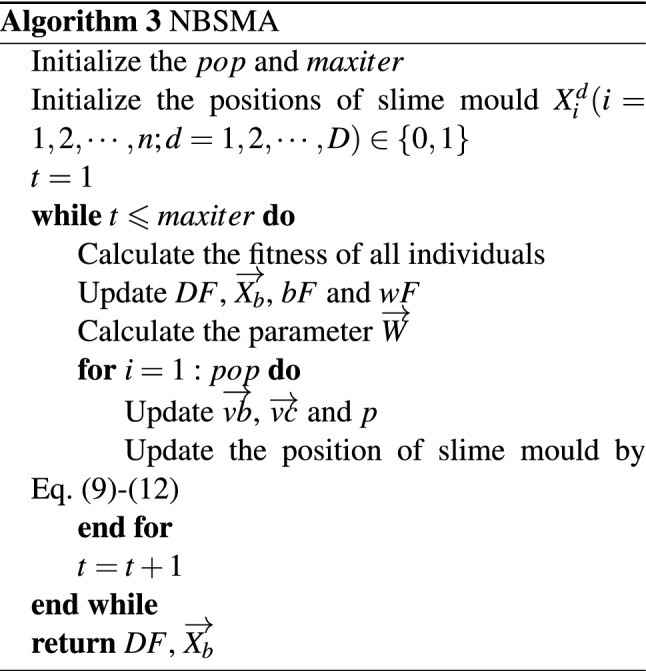



The biggest difference between NBSMA and BSMA is the position update rule when $r<p$. Table 3 shows the value of $X_{i}^{d}$ when $r<p$. It can be seen from the table that the position update process of NBSMA has nothing to do with $X_b^d$, and the update result is related to $X_b^d$.

**Table 3:** The value of $X_{i}^{d}$ when $r<p$ in NBSMA

$(X_{A}^{d},X_{B}^d)$	*Temp*	The value of $X_{i}^{d}$	
		when $F(temp)\ge rand$	when $F(temp)<rand$
(0,0)	$vb^{d}$	$1-X_b^d$	$X_b^d$
(0,1)	$-vb^{d}$	$1-X_b^d$	$X_b^d$
(1,0)	$vb^{d} \cdot W(i)$	$1-X_b^d$	$X_b^d$
(1,1)	$vb^{d} \cdot (W(i)-1)$	$1-X_b^d$	$X_b^d$

^1^*temp* has no practical meaning, but as a way to express the parameter of the transfer function

### Adding Unselected Factors Strategy (AU Strategy)

In the problem of spectrum allocation or 0-1 backpack, adding unselected factors without considering constraints will often bring better benefits. Based on this idea, this paper proposes a new strategy to mutate individuals with poor fitness. This strategy allows solutions with poor performance to increase the number of selected factors, thereby getting closer to a better solution. The pseudo-code of AU strategy is in Algorithm 4. Algorithm 5 shows the combination of the proposed NBSMA and the AU strategy.

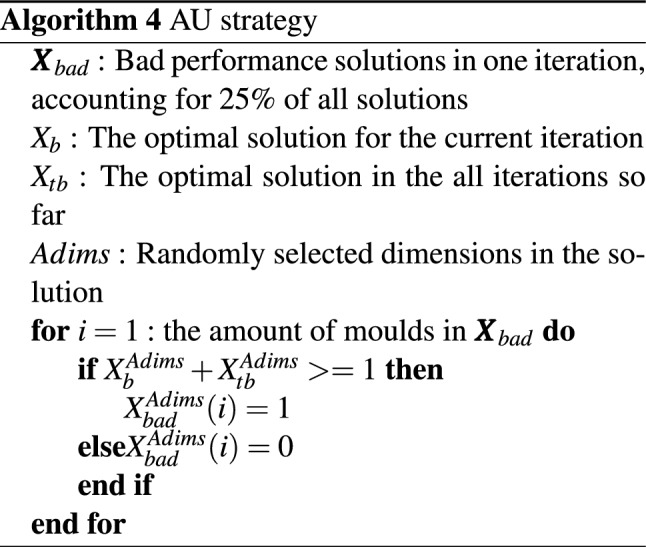

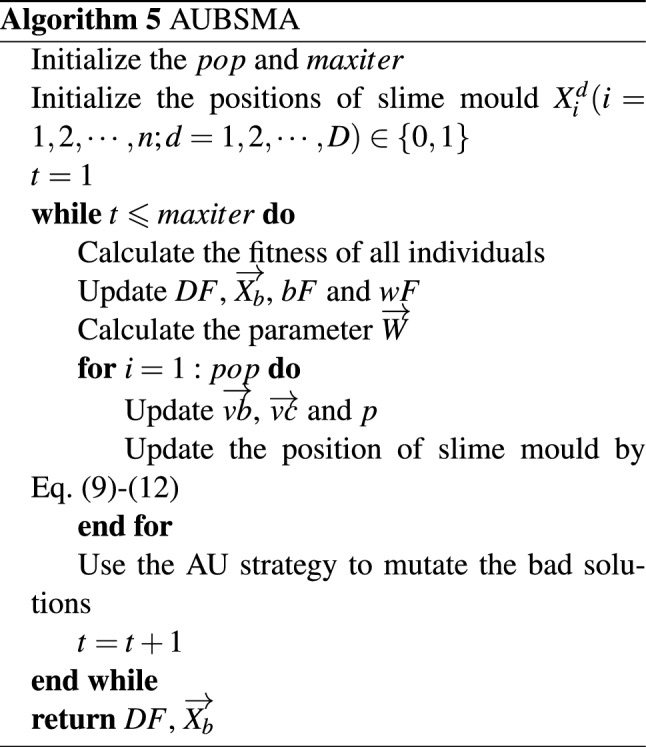





## Experimental Results and Analysis

**Fig. 2:**
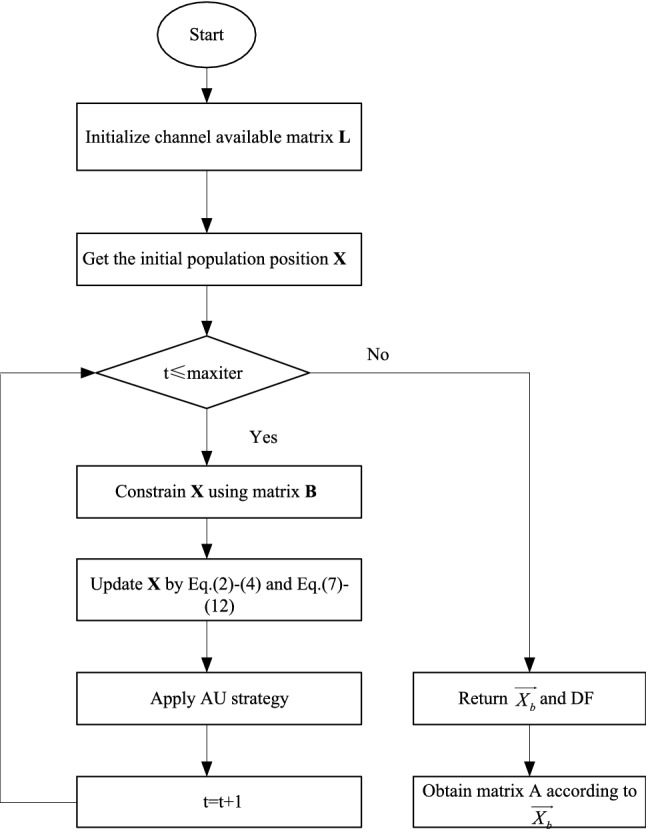
A schematic diagram of AUBSMA.

In this section, the performance of the proposed approaches is fully verified. The experiments include the verification of the transfer function, the comparison with the existing binary version of SMA and other optimization algorithms on the spectrum allocation problem, and the comparison of the running time. Take AUBSM as an example, the solution process of the algorithm will follow the process shown in Fig. 2.

### Experiments Setting

MSR and MPF represented by Eqs. (7) and  (8) are used as fitness functions in the experiments. The following assumptions are used in the experiments.

1. There are 7 situations in the cognitive network, that is, the number of channels increases from 5 to 35, increasing by 5 each time.
2. The channel environment will not change at a certain moment.
3. The number of authorized users is exactly equal to the number of channels, that is, there are no extra channels for cognitive users. Cognitive users can use the channel only when authorized users are idle.

To avoid contingency, each algorithm runs independently 30 times. There are two statistical measures of algorithm performance indicators: average (AVG) and standard deviation (STD). The parameter settings of each algorithm are shown in Table 4. In each network condition, a ranking of the performance of the algorithms is calculated. This experiment takes the solution with the greatest fitness as the optimal solution. Note that the best results will be bolded in Tables 6 to 11. The experiments are performed on a device with 8 GB of RAM and core i3 Intel CPU with 3.60 GHz and equipped with Windows 10 platform. The comparison of algorithms and the simulation of the network environment are coded by MATLAB 2017a. In the iterative optimization of the algorithms, the population size is set to 60, and the maximum iteration is 500. Each algorithm performs 30,000 evaluations on the fitness function. In order to show the details of the experiments more intuitively, Table 5 lists the related symbols summary.

**Table 4:** The parameter values of the algorithms participating in the comparison

Algorithm	Parameter settings
PSO	$\omega =0.9,c_1=c_2=2$
GA	Roulette wheel selection, crossover probability = 0.5, mutation probability = 0.6
QGA	$\alpha =\frac{1}{\sqrt{2}},\beta =\frac{1}{\sqrt{2}}$
BSMA	$z = 0.03$
AFBSMA	$z=0.03,\delta =\sigma =1.0$
FMBSMA	$z=0.03,\delta =\sigma =1.0,M_p=0.5$

**Table 5:** Summary of related symbols

Symbol	Description	Value
N	The number of cognitive users	[5:5:35]
M	The number of channels	[5:5:35]
K	The number of authorized users	[5:5:35]
Maxiter	The maximum number of iterations	500
Num	The number of runs of the algorithm under the same conditions	30
Eva	The number of evaluations for each fitness function	30000
Pop	The size of the population	60
MSR	The Max-Sum-Reward of network	
MPF	The Max-Proportional-Fair of network	

### Selection of Transfer Function

Table 6 shows the comparison of NBSMA and FMBSMA approaches on the three transfer functions of S4, S5, and V1 based on the MSR fitness function. Abdel-Basset et al. confirmed that FMBSMA is the best algorithm among the improved binary version of SMA [56]. Table 7 shows the comparison of NBSMA and FMBSMA on the three transfer functions based on MPF fitness function. Under the evaluation of the two objective functions, NBSMAS5 has the best performance in most cases. Although in the case of a small number of cognitive users ($N=5$), the performance of FMBSMAS5 is better. But with the increase in the number of cognitive users, the advantages of FMBSMAS5 disappear, and the performance of NBSMAS5 is always the best.

Among the three transfer functions of S4, S5, and V1 used by NBSMA, the performance of NBSMAS5 is always the best, followed by NBSMAV1, and the performance of NBSMAS4 is the worst. The results of FMBSMA on these three transfer functions are the same as NBSMA under the assessment of MSR.

Under the MPF evaluation, when the number of cognitive users is large ($N\le 30$), the performance ranking of FMBSMA on the three transfer functions is FMBSMAS4, FMBSMAV1, FMBSMAS5. But when the number of cognitive users is more than 5 and less than 30, the performance ranking is FMBSMAS5, FMBSMAV1, FMBSMAS4. The results of Tables 6 and  7 can draw the conclusion that the S5 transfer function is the best in the NBSMA algorithm and the FMBSMA algorithm. And the performance of NBSMAS5 is better than FMBSMAS5.

**Fig. 3:**
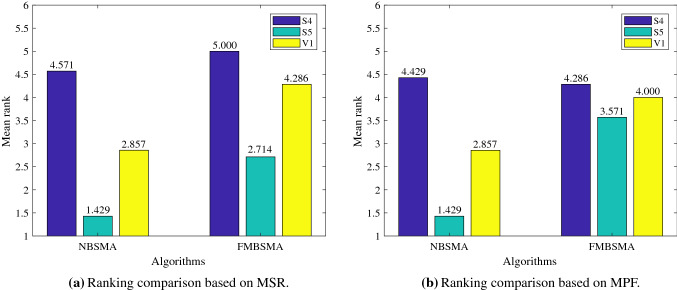
The mean of the algorithm rankings under different network conditions

Figure 3 shows the mean of the rankings of the algorithms in the seven cases in the comparison results of Tables 6 and  7. Figure 3 can further support the above analysis and prove that the S5 transfer function has better performance in both algorithms.

**Fig. 4:**
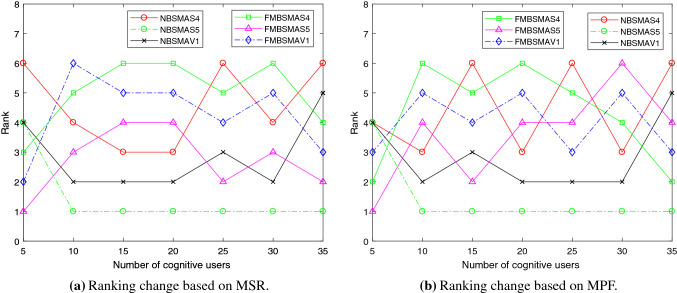
The impact of the number of cognitive users on the performance of the algorithm

Figure 4 shows the ranking changes of the six approaches with the increase in cognitive users under the two fitness functions. It can be clearly seen that when the number of cognitive users is 5, FMBSMAS5 has the best performance under the two objective functions of network reward and fairness. But as the number of cognitive users increases, NBSMAS5 has the best performance. In subsequent experiments, S5 will be used as the transfer function.

**Table 6:** Comparison results based on MSR

Number of users		NBSMAS4	NBSMAS5	NBSMAV1	FMBSMAS4	FMBSMAS5	FMBSMAV1
N=5	AVG	98.341	98.442	98.442	143.633	**144.216**	143.696
	STD	0.071	0	0	0.868	0	0.82
	Rank	6	4	4	3	1	2
N=10	AVG	309.688	**382.17**	381.6	280.99	311.306	280.487
	STD	7.609	0	0.779	5.42	7.126	7.348
	Rank	4	1	2	5	3	6
N=15	AVG	673.219	**909.388**	876.98	601.578	658.572	615.986
	STD	10.704	0.91	3.464	11.673	8.817	9.711
	Rank	3	1	2	6	4	5
N=20	AVG	874.387	**1291.756**	1081.238	613.106	688.821	640.89
	STD	8.445	7.906	28.304	9.054	6.18	7.782
	Rank	3	1	2	6	4	5
N=25	AVG	951.813	**1459.496**	1156.324	1064.78	1161.149	1105.425
	STD	16.427	6.142	18.503	16.638	16.471	19.06
	Rank	6	1	3	5	2	4
N=30	AVG	1312.257	**2064.413**	1576.641	1269.312	1359.555	1297.288
	STD	11.287	24.174	28.789	11.814	17.716	13.894
	Rank	4	1	2	6	3	5
N=35	AVG	1283.737	**2011.188**	1492.192	1632.821	1662.477	1633.493
	STD	16.241	19.843	20.425	19.005	19.347	6.739
	Rank	6	1	5	4	2	3
Mean rank		4.571	1.429	2.857	5.000	2.714	4.286

**Table 7:** Comparison results based on MPF

Number of users		NBSMAS4	NBSMAS5	NBSMAV1	FMBSMAS4	FMBSMAS5	FMBSMAV1
N=5	AVG	12.55	12.55	12.55	18.353	**19.246**	17.855
	STD	0	0	0	0.379	0	0.55
	Rank	4	4	4	2	1	3
N=10	AVG	20.207	**23.335**	22.999	16.569	19.238	17.445
	STD	0.499	0.026	0.231	0.518	0.339	0.293
	Rank	3	1	2	6	4	5
N=15	AVG	25.733	**33.584**	31.037	28.49	32.933	31.032
	STD	0.177	0.05	0.099	0.545	0.346	0.306
	Rank	6	1	3	5	2	4
N=20	AVG	31.509	**41.14**	35.595	22.244	23.666	22.782
	STD	0.106	0.296	0.638	0.601	0.778	0.226
	Rank	3	1	2	6	4	5
N=25	AVG	30.303	**40.356**	32.779	31.64	31.653	31.948
	STD	0.197	0.201	0.769	0.309	0.303	0.451
	Rank	6	1	2	5	4	3
N=30	AVG	32.07	**45.381**	35.434	29.498	28.633	29.017
	STD	0.285	0.328	1.028	0.251	0.233	0.58
	Rank	3	1	2	4	6	5
N=35	AVG	28.587	**37.767**	30.082	35.085	34.03	34.188
	STD	0.359	0.196	0.315	0.146	0.575	0.796
	Rank	6	1	5	2	4	3
Mean rank		4.429	1.429	2.857	4.286	3.571	4.000

### Comparison of the Proposed Algorithms with Binary Versions of SMA

In this section, the proposed NBSMA and AUBSMA are compared with the three versions of binary SMA (BSMA, AFBSMA, and FMBSMA) proposed by Mohamed Abdel-Basset et al. These approaches all use the S5 transfer function to convert continuous values.

Table 8 shows the comparison results based on MSR of the network. Table 9 shows the comparison results based on MPF. From Table 8, it can be seen that AUBSMA using the AU strategy has better performance overall. When there are few cognitive users ($N=5$), the performance of NBSMA, AUBSMA, FMBSMA, and AFBSMA is the same. When the number of cognitive users is 15 or 20, NBSMA has the best performance, but the performance of the AUBSMA is only slightly worse than NBSMA. The values of the two approaches on AVG are very close. In other cases, the performance of AUBSMA is the best. In Table 9, when there are few cognitive users ($N\le 10$), the advantages of NBSMA and AUBSMA are not obvious. NBSMA has the best performance when the number of cognitive users reaches 20. In other cases, the proposed AUBSMA approach has the best performance. With the increase in users, the gap between AUBSMA and other algorithms increases.

**Fig. 5:**
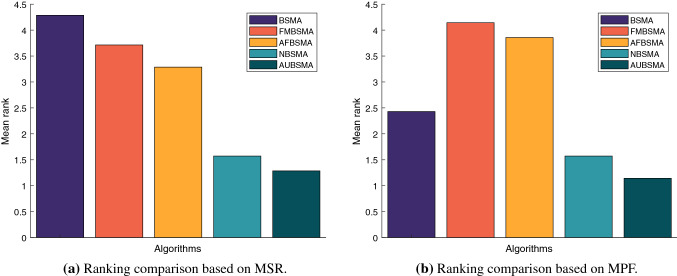
The mean of the algorithm rankings under different network conditions

Figure 5 depicts the mean of rankings under the two objectives of network reward and fairness. It can be seen that the performance of AUBSMA is the best, followed by NBSMA.

**Fig. 6:**
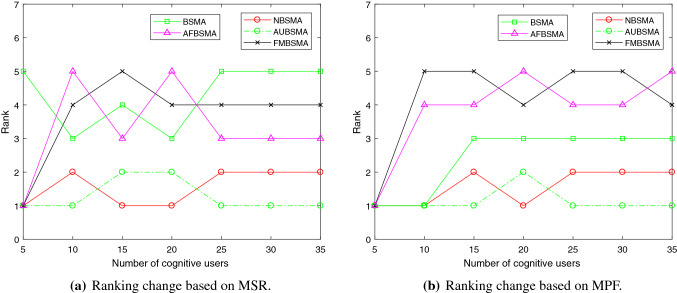
The impact of the number of cognitive users on the performance of the algorithms

Figure 6 shows how the ranking of the algorithm changes as the number of cognitive users increases under the two fitness functions. In the comparison based on MSR shown in Fig. 6a, when the number of cognitive users reaches 25, the performance of each algorithm tends to stabilize, and the ranking no longer changes. In the comparison based on MPF, when the number of cognitive users is 5, the five versions of the binary SMA algorithms have the same performance.

**Fig. 7:**
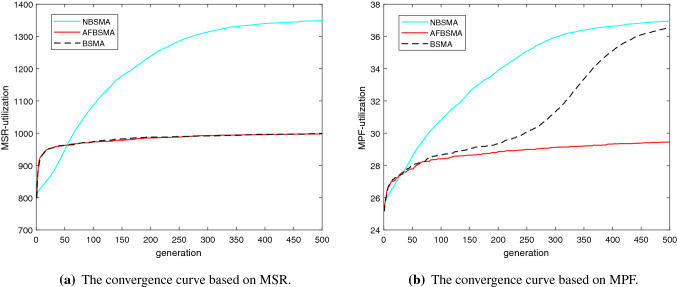
The convergence curves of NBSMA, AFBSMA and BSMA

Figure 7 shows the comparison of the convergence curves of NBSMA with AFBSMA and BSMA. It can be seen from Tables 8 and  9 that that among the existing binary SMA methods, AFBSMA and BSMA perform best on MSR and MPF, respectively. The convergence curves are the average MSR and MPF under seven network conditions. It can be found from Fig. 7a that NBSMA does not converge prematurely. After reaching 100 iterations, the convergence speed of NBSMA is slower than before, indicating that it has entered the local search stage. However, BSMA and AFBSMA stopped converging at an early stage. Figure 7b shows the convergence curves of the three algorithms on MPF. Because it is different from the fitness function in Fig. 7a, BSMA shows a different convergence effect than in MSR. Although AFBSMA applies the two-phase mutation strategy [56], its convergence performance is worse than BSMA. As can be seen from the figure, NBSMA is able to maintain a balance between exploration and exploitation.

**Table 8:** Comparison of different binary versions of SMA algorithm based on MSR

Number of users		BSMA [56]	FMBSMA [56]	AFBSMA [56]	NBSMA	AUBSMA
N=5	AVG	120.353	**120.452**	**120.452**	**120.452**	**120.452**
	STD	0.221	0	0	0	0
	Rank	5	1	1	1	1
N=10	AVG	430.199	428.744	425.573	470.616	**471.132**
	STD	9.022	3.727	2.037	4.245	4.085
	Rank	3	4	5	2	1
N=15	AVG	613.239	609.973	617.677	**698.703**	698.339
	STD	7.492	7.928	5.742	4.721	5.085
	Rank	4	5	3	1	2
N=20	AVG	849.481	836.473	832.814	**1102.55**	1099.302
	STD	8.527	9.156	12.776	4.164	7.065
	Rank	3	4	5	1	2
N=25	AVG	863.849	869.413	882.83	1439.819	**1452.21**
	STD	8.682	18.237	11.175	14.537	14.084
	Rank	5	4	3	2	1
N=30	AVG	1464.133	1469.24	1482.884	2182.988	**2193.65**
	STD	17.313	13.203	24.005	20.045	19.17
	Rank	5	4	3	2	1
N=35	AVG	1528.96	1539.376	1548.572	2135.067	**2207.33**
	STD	28.008	20.651	23.234	14.448	17.535
	Rank	5	4	3	2	1
Mean rank		4.286	3.714	3.571	1.571	1.286

**Table 9:** Comparison of different binary versions of SMA algorithm based on MPF

Number of users		BSMA [56]	FMBSMA [56]	AFBSMA [56]	NBSMA	AUBSMA
N=5	AVG	**11.202**	**11.202**	**11.202**	**11.202**	**11.202**
	STD	0	0	0	0	0
	Rank	1	1	1	1	1
N=10	AVG	**29.979**	28.753	29.007	**29.979**	**29.979**
	STD	0	0.533	0.502	0	0
	Rank	1	5	4	1	1
N=15	AVG	27.595	24.771	24.804	27.852	**27.93**
	STD	0.207	0.362	0.621	0.148	0.191
	Rank	3	5	4	2	1
N=20	AVG	37.142	31.029	30.71	**37.431**	37.168
	STD	0.21	0.238	0.225	0.203	0.303
	Rank	3	4	5	1	2
N=25	AVG	34.599	23.591	24.382	35.134	**35.315**
	STD	0.091	0.389	0.412	0.211	0.251
	Rank	3	5	4	2	1
N=30	AVG	47.965	32.161	32.61	49.711	**50.148**
	STD	0.472	0.655	1.018	0.362	0.264
	Rank	3	5	4	2	1
N=35	AVG	45.727	32.597	32.115	47.284	**49.513**
	STD	0.375	1.07	0.625	0.229	0.388
	Rank	3	4	5	2	1
Mean rank		2.429	4.143	3.857	1.571	1.143

### Comparison of the Proposed Algorithms with Other Binary Algorithms

In this section, the proposed approaches of NBSMA and AUBSMA are compared with PSO [59], GA [7], and QGA [7] that have been used to solve the spectrum allocation. Tables 10 and 11 show the comparison results under the two objective functions of MSR and MPF. The results show that in the comparison of five algorithms, AUBSMA has the best performance, and the performance of NBSMA is slightly worse than that of AUBSMA. When the number of cognitive users is 10 or 15, NBSMA outperforms AUBSMA in fairness. In most cases, the performance of GA is the worst. When the number of cognitive users is large, the performance of PSO is slightly better than QGA.

**Fig. 8:**
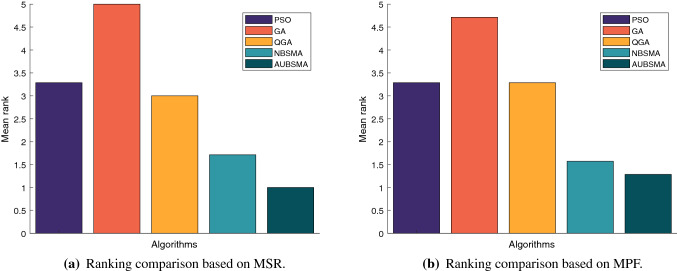
The mean of the algorithm rankings under different network conditions

Figure 8 depicts the mean of the ranking of the algorithm in seven situations under the two objective functions of MSR and MPF. It can be seen that AUBSMA has the best performance.

**Fig. 9:**
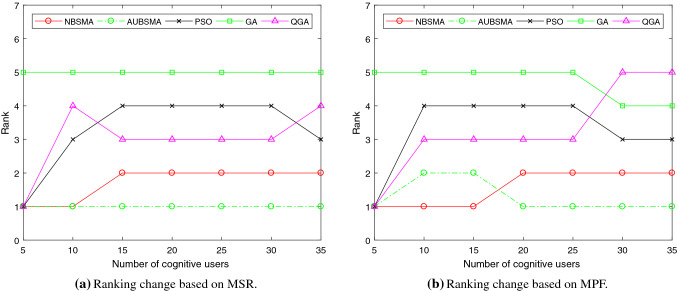
The impact of the number of cognitive users on the performance of the algorithms

Figure 9 shows the changes in the ranking of the algorithm with the increase in the number of cognitive users under the two fitness functions. Figure 9a shows that the performance of AUBSMA has always been better under the comparison based on the MSR. When the number of cognitive users is 10, NBSMA has the same excellent performance as AUBSMA. But when the number of cognitive users is more than 10, the performance of NBSMA is worse than AUBSMA. The performance of GA has always been the worst. In the comparison based on fairness shown in Fig. 9b, NBSMA is better than AUBSMA in a few cases. But when the number of cognitive users increases and the AU strategy has played a role, AUBSMA performs best.

**Table 10:** Comparison of the algorithms based on MSR

Number of users		PSO [59]	GA [7]	QGA [7]	NBSMA	AUBSMA
N=5	AVG	**161.471**	154.747	**161.471**	**161.471**	**161.471**
	STD	0	2.362	0	0	0
	Rank	1	5	1	1	1
N=10	AVG	349.671	284.61	346.285	**370.11**	**370.11**
	STD	22.924	6.025	9.248	0	0
	Rank	3	5	4	1	1
N=15	AVG	418.994	373.263	426.168	505.478	**508.091**
	STD	11.853	8.002	5.528	4.495	4.315
	Rank	4	5	3	2	1
N=20	AVG	1017.369	906.481	1019.816	1324.409	**1326.21**
	STD	14.299	11.529	13.243	11.967	7.519
	Rank	4	5	3	2	1
N=25	AVG	1072.579	977.19	1097.755	1389.101	**1394.7**
	STD	25.914	9.718	6.837	7.699	7.986
	Rank	4	5	3	2	1
N=30	AVG	1429.013	1306.198	1466.033	2099.251	**2119.57**
	STD	40.183	21.948	14.203	14.402	10.996
	Rank	4	5	3	2	1
N=35	AVG	1598.815	1508.462	1524.729	2305.389	**2365.94**
	STD	16.626	11.193	17.499	20.679	20.141
	Rank	3	5	4	2	1
Mean rank		3.286	5.000	3.000	1.714	1.000

**Table 11:** Comparison of the algorithms based on MPF

Number of users		PSO [59]	GA [7]	QGA [7]	NBSMA	AUBSMA
N=5	AVG	**27.429**	27.122	**27.429**	**27.429**	**27.429**
	STD	0	0.647	0	0	0
	Rank	1	5	1	1	1
N=10	AVG	22.399	19.692	23.152	**24.334**	24.061
	STD	0.993	0.368	0.434	0.65	0.582
	Rank	4	5	3	1	2
N=15	AVG	22.76	20.965	24.179	**27.575**	27.498
	STD	0.372	0.557	0.582	0.168	0.156
	Rank	4	5	3	1	2
N=20	AVG	36.206	34.772	37.168	42.885	**42.914**
	STD	0.807	0.358	0.269	0.214	0.411
	Rank	4	5	3	2	1
N=25	AVG	29.642	28.006	30.361	36.516	**36.725**
	STD	0.666	0.265	0.42	0.13	0.152
	Rank	4	5	3	2	1
N=30	AVG	32.423	31.384	31.078	43.25	**43.97**
	STD	0.281	0.284	0.292	0.303	0.462
	Rank	3	4	5	2	1
N=35	AVG	32.931	31.949	30.788	42.317	**43.84**
	STD	0.162	0.568	0.691	0.497	0.344
	Rank	3	4	5	2	1
Mean rank		2.429	4.143	3.857	1.571	1.143

### Comparison of the Running Time of Algorithms

**Fig. 10:**
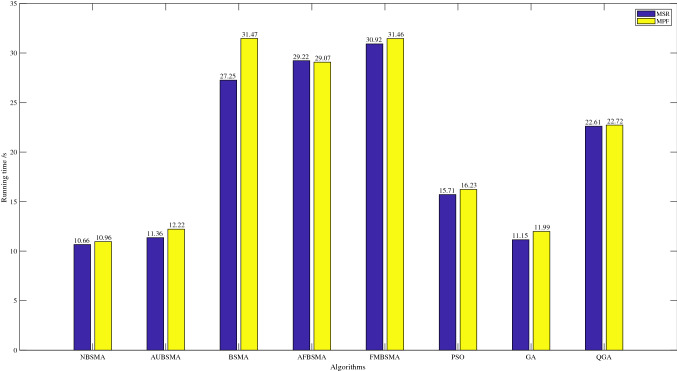
Comparison of the running time of different optimization algorithms

Figure 10 shows the comparison of the running time of eight algorithms on the two goals of MSR and MPF. In this comparison, the network environment is set to the situation when the number of channels is 20$(M=20)$. According to the assumptions mentioned in Sect. 4.1, the number of authorized users and cognitive users is also 20. The maximum number of iterations and the number of independent runs of the algorithm are also described in Sect. 4.1. It can be seen that under the same conditions, the shortest running time is NBSMA, followed by GA and AUBSMA. The reason why AUBSMA runs slightly longer than NBSMA is that it further applies the AU strategy on the basis of NBSMA.

## Conclusion

In this work, NBSMA is proposed to solve the scheme of spectrum allocation. A transfer function that has not been used in BSMA algorithm is added to the comparison. The newly added transfer function is compared with the better two of the S-shaped and V-shaped transfer functions. Experiments prove that the newly added transfer function has a relatively superior effect. For agents that perform poorly in the search process, this paper proposes the strategy of adding unselected factors to mutate the solutions. AUBSMA, which combines NBSMA with this strategy, can achieve more superior performance than NBSMA. In terms of the goals of MSR and MPF, NBSMA and AUBSMA proposed in this paper are superior to other optimization algorithms, such as BSMA, FMBSMA, AFBSMA, PSO, QGA, and GA in most network situations.

The two approaches proposed in this paper have only been verified on spectrum allocation and have not been compared with numerous binary optimization algorithms on the benchmark platform. For future work, studying a dynamically changing transfer function may be very helpful to improve the performance of the binary optimization problem. In addition, the proposed approaches can be used to try to solve problems such as knapsack problems, feature selection, or fault location of the distribution network.

## Data Availability

The data used to support the findings of this study are included in the article.
